# Assessment of the effects of prepartum anti-inflammatory therapies on type 1/type 2 immunity ratio using a rapid blood test

**DOI:** 10.3168/jdsc.2023-0444

**Published:** 2023-11-17

**Authors:** J. Spring, Q. Huo, E. Jimenez, M. Martinez, P. Zarei, J. Lection, E. Hovingh, J. Lawhead, R.H. Sorto Cruz, A.A. Barragan

**Affiliations:** ^1^ Department of Veterinary and Biomedical Sciences, The Pennsylvania State University, University Park, PA 16802; ^2^ Department of Chemistry and NanoScience Technology Center, University of Central Florida, Orlando, FL 32826; ^3^ Intergraduate Degree Program in Integrative and Biomedical Physiology, Huck Institutes of the Life Sciences, The Pennsylvania State University, University Park, PA 16802; ^4^ Millerstown Veterinary Associates, Millerstown, PA 17062

## Abstract

The objective of this study was to assess (1) the effects of prepartum administration of anti-inflammatory therapies on type 1/type 2 immunity ratio using a rapid blood test (D2Dx immunity test; Nano Discovery Inc.), and (2) correlations between rapid blood test scores and daily milk yield in Holstein dairy cows. At 14 d before the expected calving date, cows (n = 64) and heifers (n = 23) were blocked by body condition score (optimal = 3.25–3.5; high ≥3.75) and parity (nulliparous, parous), and randomly allocated to one of 3 treatment groups (1) ASA (n = 29) = receive one oral treatment with administration of acetylsalicylic acid (4 boluses; 480 grain/bolus); (2) MEL (n = 31) = receive one oral administration with meloxicam (1 mg/kg of body weight), or (3) PLC (n = 27) = receive one oral treatment with 4 gelatin capsules filled with water. Blood samples were collected weekly starting 1 wk before treatment until 3 wk after calving for assessment of type 1/type 2 immunity ratio using a rapid blood test (i.e., D2Dx immunity test). A higher D2Dx score corresponds to a higher type 1/type 2 ratio. Furthermore, blood samples were collected within 72 h before and after calving by farm personnel. Daily milk yield for the first 60 d in milk (DIM) was collected from on-farm computer records. The data were analyzed using MIXED procedure of SAS (SAS Institute Inc.) as a randomized complete block design. On average enrolled cows received treatment administration 10 d before the actual calving date (standard deviation = 5.10 d). There was a tendency for a treatment by day interaction. Cows treated with ASA had higher type 1/type 2 ratio within 3 d after calving compared with MEL and PLC cows (ASA = 0.065 ± 0.002; MEL = 0.059 ± 0.002; PLC = 0.053 ± 0.002). Similarly, ASA and MEL cows had a higher type 1/type 2 ratio at 7 ± 3 DIM compared with PLC cows (ASA = 0.062 ± 0.002; MEL = 0.064 ± 0.002; PLC = 0.056 ± 0.002). Regardless of treatment, there was an interaction between parity and day. Parous cows had higher type 1/type 2 ratios compared with nulliparous cows at 14 ± 3 d before calving and at 7 ± 3, 14 ± 3, and 21 ± 3 d after calving. Furthermore, there was a positive correlation between D2Dx scores at 14 ± 3 DIM and average daily milk yield in the first 60 DIM. These results suggest that prepartum anti-inflammatory therapies may cause an increased shift in type 1 immunity around calving. Similarly, parous cows may have an increased shift in type 1 immunity after calving. Interestingly, higher type 1/type 2 ratios may be associated with higher milk yields in the first 60 DIM. Larger studies are needed to identify associations between the D2Dx immunity test and cow health and performance, as well as to assess the applicability of these types of tests in a conventional farm setting.

## Highlights

• Prepartum anti-inflammatory therapies may increase type 1 immunity shift.
• Parous cows may have an increased shift in type 1 immunity after calving.
• A higher type 1/type 2 ratio might be associated with higher milk yields.
• Larger studies are needed to assess associations between this test and cow health.

## Graphical Abstract

**Figure:**
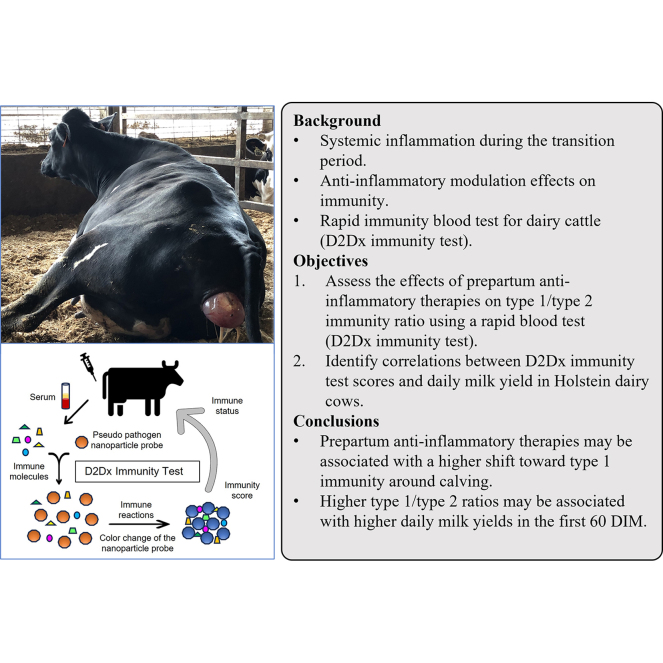
**Summary** During the transition period, cows experience systemic inflammatory processes necessary for physiological events related to calving; however, an exacerbated inflammatory response could increase the risk of developing diseases and poor performance. Postpartum anti-inflammatory treatments have been proposed as an alternative to modulating this inflammatory response. Moreover, prepartum anti-inflammatory strategies have not been investigated yet. The objective of this study was to assess the effects of prepartum administration of anti-inflammatory therapies on type 1/type 2 immunity ratio using a rapid blood test (D2Dx immunity test) and identify correlations between the rapid blood test scores and daily milk yield in Holstein dairy cows. Cows treated with acetylsalicylic acid and meloxicam had higher type 1/type 2 immunity ratios around calving compared with placebo animals. These results suggest that prepartum anti-inflammatory therapies may cause an increased shift in type 1 immunity around calving, which may be associated with stronger immunity.

The peri-parturient period, also known as the transition period, encompasses the 3 wk before and the 3 wk after calving. This is a crucial time for dairy cows when major nutritional, metabolic, and immunological changes challenge their health and performance (Drackley, 1999; Sordillo and Raphael, 2013). During this time, cows also experience systemic inflammatory processes necessary for physiological events related to calving. However, an exacerbated inflammatory response could increase the risk of experiencing postpartum diseases, having poor productivity and reproductive performance, and being culled (Bradford et al., 2015). Anti-inflammatory treatments, such as the use of nonsteroidal anti-inflammatory drugs (**NSAIDs**), have been proposed as an alternative to modulate inflammation after calving (Carpenter et al., 2018; Swartz et al., 2018; Barragan et al., 2020a,b,c).

During gestation, reproductive hormones (e.g., progesterone) and the conceptus (IFNτ and other signaling factors) regulate the maternal immune system, mainly causing a depression in the immune response aimed at preventing nonrecognition of, and a following immune response against, the developing fetus (Vlasova and Saif, 2021). This immune balance is characterized by a shift toward type 2-biased immunity in the cow adaptative immune response, which is characterized by a larger Th2 cell response, increased monocytes and dendritic cells in the endometrial stroma, and M2 activated macrophages, which secrete cytokines (e.g., IL-4, IL-10, TGF-b) to decrease activation of anti-conceptus immunity (Vlasova and Saif, 2021). It has been suggested that cows experience a shift from type 2-biased immunity to type 1-biased immunity shortly after calving (Tsai et al., 2021). In this immune balance, there is a larger response of Th1 cells, which is targeted to protect the animal against infectious agents instead of creating immune tolerance for the developing fetus (Vlasova and Saif, 2021). In general, type 1-biased immunity may have a more protective effect against most common pathogens compared with type 2-biased immunity, especially against intracellular pathogens such as viruses and intracellular bacteria (Spellberg and Edwards, 2001). This immunity shift around parturition is further driven by the removal of IgG1, associated with a more potent type 2-biased immune response, from the blood circulation for colostrum production (Vlasova and Saif, 2021).

The D2Dx immunity test (Nano Discovery Inc.) is a rapid blood test that is used to measure the humoral immune status in farm animals (Zheng et al., 2019, 2020; Deb et al., 2021; Tsai et al., 2021). The test detects the humoral immune response of a blood serum sample in the presence of a nanoparticle pseudo-pathogen probe in vitro. A collective interaction of major immune molecules including IgG, IgM, and complement proteins with the nanoparticle pseudo-pathogen probe leads to a color change of the nanoparticle solution, and this color change is detected and correlated with the humoral immune status of the animal (Zheng et al., 2019). Furthermore, the nanoparticle probe is designed to distinguish type 1 versus type 2 immunity based on the differential interaction between type 1 immunity-related IgG subclasses and type 2 immunity-related IgG subclasses with the nanoparticle probe (Zheng et al., 2020; Deb et al., 2021; Tsai et al., 2021). A type 1-biased immunity leads to higher D2Dx scores, whereas a type 2-biased immunity leads to lower D2Dx scores. This test has been validated in farm animals, including cattle. Zheng et al. (2020) assessed D2Dx scores in cows in different stages of the gestation, and reported that as cows got closer to calving, they had higher D2Dx scores. Authors also reported that scores peaked just around calving and decreased afterward (Zheng et al., 2020).

The objective of this study was to the assess (1) effects of prepartum administration of anti-inflammatory therapies on type 1/type 2 immunity ratio using a rapid blood test (D2Dx immunity test), and (2) correlations between this rapid blood test scores and daily milk yield in Holstein dairy cows. We hypothesized that cows treated with the proposed anti-inflammatory protocols will have higher type 1-biased immunity reflected by higher D2Dx test scores, and animals with higher D2Dx test score will be correlated with daily milk yield.

Dairy cows from a dairy farm (700 milking cows) located in central Pennsylvania were enrolled in this study. The yearly rolling herd average milk yield was 11,143 kg. Cows and heifers were moved to a close-up pen at 14 ± 3 d before their expected calving date and housed together in a loose pen with deep straw bedding until calving. After calving, primiparous cows and multiparous were housed together into a postpartum pen until approximately 4 to 5 d after calving. Then, cows were moved to larger pens where they remained for the rest of their lactation. Animals were housed in naturally ventilated freestall pens with deep sand bedding, equipped with additional fans and sprinklers. Cows were milked 3 times a day, approximately 8 h apart, and fed a TMR diet formulated to meet or exceed dietary nutritional requirement (NASEM, 2021) twice daily. All the procedures described below were approved by the Institutional Animal Care and Use Committee at The Pennsylvania State University (Protocol number 202101971).

Study animals were enrolled from August 2021 until January 2022. Briefly, a list of dry cows that were between 265 and 271 d in gestation was obtained weekly by the research team from on-farm computer records. Dry cows (n = 64) and heifers (n = 23) were blocked by BCS (optimal = 3–3.5; high ≥3.75) and parity (nulliparous [**NUL**]; parous [**PAR**]), and randomly allocated (RAND function, Microsoft Excel, 2017, Microsoft Corp.) by the study team to one of 3 treatment groups: (1) **ASA** (n = 29) = receive one oral treatment with administration of acetylsalicylic acid (4 boluses; 480 grain/bolus; Sparhawk Laboratories Inc., KS) 14 d before expected calving date, (2) **MEL** (n = 31) = receive one oral administration with meloxicam (1 mg/kg of BW; Zydus Pharmaceutical, NJ) 14 d before expected calving date, or (3) **PLC** (n = 27) = receive one oral treatment with 4 gelatin capsules filled with water 14 d before expected calving date. Animals where restrained using feed bunk headlocks and the research team administered all the treatments. Due to the logistics of preparing the gelatin capsules for the PLC group (capsules dissolve in a few minutes after adding water), the research team was not blinded when administrating treatment.

Blood samples were collected into a 10­-mL Vacutainer tubes (BD Vacutainer, Becton Dickinson and Company, Franklin Lakes, NJ) weekly starting 1 wk before treatment until 3 wk after calving by the study team. Furthermore, blood samples were collected within 72 h before and after calving by farm personnel. Samples were kept on ice until they were centrifuged (within ∼2 h) for 15 min at 1,400 × *g* at room temperature (25°C) to collect serum. Serum was frozen at −20°C until further analysis. To perform the D2Dx test, 10 µL of blood serum sample was mixed with 50 µL of nanoparticle probe solution in a mini-cuvette. After vortex mixing for 5 s, the mini-cuvette was placed in the sample holder of a test reader device, CT-100 (Nano Discovery Inc.). The color change of the assay solution within a 30 s of reaction time was recorded as the test score. Daily milk yield for the first 60 DIM was collected using parlor meters (SCR Dairy, Netanya, Israel). The average daily milk yield (kg/d) at 60 DIM was calculated by averaging the individual daily milk yield averages for the first 60 DIM.

To be able to detect a difference of 0.005 in absorbance (healthy vs. diseased groups at 14 DIM; Tsai et al., 2021) between study groups, with adequate statistical power (1 − β = 0.8) and significance (α = 0.05), a sample size of 16 dairy cows per group (total of 48 animals) was required. This randomized complete block design was analyzed using the SAS statistical software (version 9.4, SAS Institute Inc., Cary, NC). The UNIVARIATE procedure of SAS was used to assess the homogeneity and normality of variances (graphical method, such as histogram and quantile-quantile plot, and Bartlett's tests; Shapiro-Wilk statistic) for the quantitative variables. The D2Dx test scores were analyzed using the MIXED procedure of SAS. The REPEATED statement was included in the MIXED procedure. In addition, because the first sample (i.e., −14 ± 3 d before calving) was collected immediately before treatment administration, this value was forced in the model as covariate to be used as baseline data between treatment groups (Montgomery et al., 2019). The covariate structures were selected using the best fit according to Schwarz's Bayesian information criteria. The variables that remained in the model were selected using the Wald statistic backward selection criterion (*P* > 0.15). The variable cow was included in the RANDOM statement of the MIXED procedure. The variable treatment, day, and day by treatment interaction (where the REPEATED statement was used) were forced in the models. The results are presented as least squares means and standard error of the mean, calculated and adjusted with Tukey-Kramer method using the LSMEAN statement. When the interaction between treatment and day or other variable was significant (*P* < 0.05), the “slice” option in the “lsmeans” statement was used to determine differences among treatments on each level (single comparison) of the interacting variable. The main variables of interest and their interactions were considered significant if *P* < 0.05, and 0.05 < *P* ≤ 0.10 was considered a tendency. Pearson correlation coefficients were computed using the PROC CORR procedure in SAS for the correlation analysis between D2Dx test scores and average daily milk yield in the first 60 DIM.

Eighty-eight Holstein dairy cows were enrolled in this study. Sixty-four were PAR animals (ASA = 22; MEL = 22; PLC = 20) and 24 were NUL (ASA = 7; MEL = 9; PLC = 8). On average enrolled cows received treatment administration at 10 d before actual calving date (SD = 5.10 d). There was a tendency (*P* = 0.09) for a treatment by day interaction. Cows treated with MEL tended (*P* = 0.09) to have a higher type 1/type 2 ratio within 72 h before calving (ASA = 0.051 ± 0.004; MEL = 0.057 ± 0.004; PLC = 0.045 ± 0.005; Figure 1), whereas ASA cows had (*P* = 0.005) higher type 1/type 2 ratio within 3 d after calving compared with MEL and PLC cows (ASA = 0.065 ± 0.002; MEL = 0.059 ± 0.002; PLC = 0.053 ± 0.002; Figure 1). Similarly, ASA and MEL cows had (*P* = 0.04) a higher type 1/type 2 ratio at 7 ± 3 DIM compared with PLC cows (ASA = 0.062 ± 0.002; MEL = 0.064 ± 0.002; PLC = 0.056 ± 0.002; Figure 1). Regardless of treatment, there was an interaction between parity and day (*P* = 0.005), where parous cows had higher type 1/type 2 ratios compared with nulliparous cows at −14 ± 3 d before calving and at 7 ± 3, 14 ± 3, and 21 ± 3 d after calving (Table 1). Daily milk yield data for the first 60 DIM were available for a total of 69 cows (ASA NUL = 5; ASA PAR = 19; MEL NUL = 5; MEL PAR = 19; PLC NUL = 4; PLC PAR = 20). There was a positive weak correlation (r = 0.31) between D2Dx scores at 14 ± 3 DIM and daily milk yield in the first 60 DIM (Figure 2).

**Figure 1:**
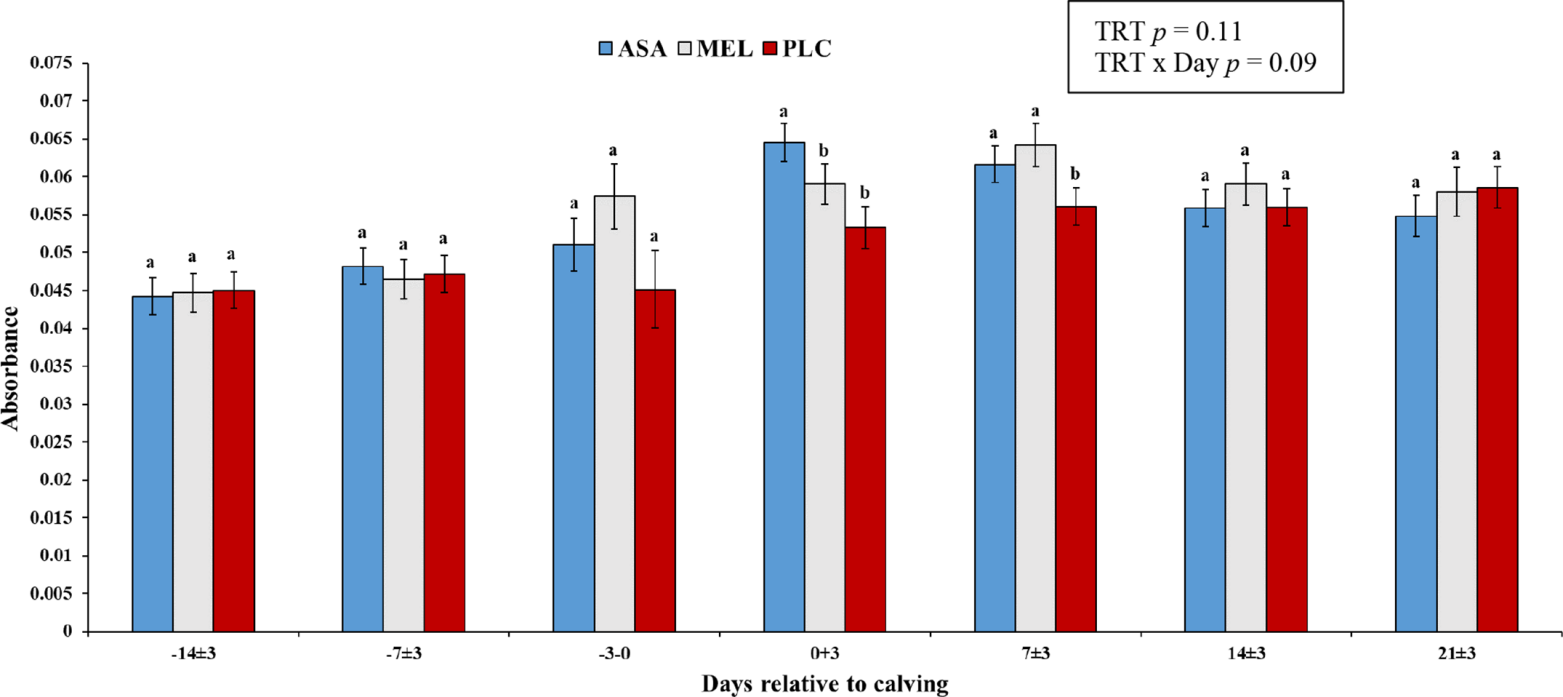
D2Dx immunity test (Nano Discovery Inc.) absorbance values in cows treated with acetylsalicylic acid (ASA, n = 29), meloxicam (MEL, n = 31), or a placebo (PLC, n = 28) 14 d before the expected calving date. Values are presented as LSM ± SEM. Different letters (a, b) indicate significant differences (*P* < 0.05). TRT = treatment.

**Table 1:** D2Dx immunity test (Nano Discovery Inc.) absorbance values (LSM ± SEM) in nulliparous (n = 24) and parous (n = 64) cows treated with acetylsalicylic acid, meloxicam, or a placebo 14 d before the expected calving date

Parity^1^	Days relative to calving							Overall
	−14 ± 3	−7 ± 3	−3–0	0+3	7 ± 3	14 ± 3	21 ± 3	
Nulliparous	0.046 ± 0.003^a^	0.045 ± 0.003^a^	0.042 ± 0.004^a^	0.053 ± 0.003^a^	0.055 ± 0.003^a^	0.052 ± 0.003^a^	0.052 ± 0.004^a^	0.049 ± 0.002^a^
Parous	0.044 ± 0.002^a^	0.050 ± 0.002^a^	0.060 ± 0.003^b^	0.065 ± 0.002^b^	0.067 ± 0.002^b^	0.062 ± 0.002^b^	0.062 ± 0.002^b^	0.058 ± 0.001^b^

a,b Different letters within a column indicate a significant difference (*P* < 0.05).

1 Nulliparous: prepartum heifers (lactation = 0). Parous: cows that have calved at least once (lactation ≥1).

**Figure 2:**
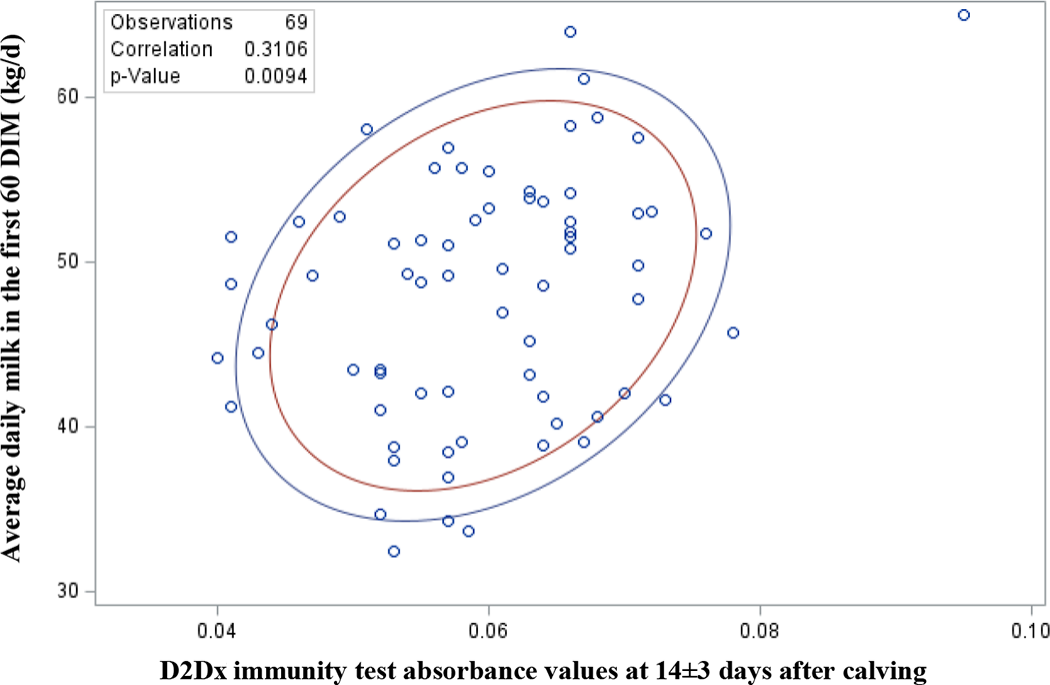
Scatter plot showing the correlation between D2Dx immunity test (Nano Discovery Inc.) absorbance values at 14 ± 3 d after calving and average daily milk yield for the first 60 DIM in cows treated with acetylsalicylic acid (ASA, n = 29), meloxicam (MEL, n = 31), or a placebo (PLC, n = 29) 14 d before the expected calving date. The prediction ellipses approximate a region that contains a specified percentage of the population (70% and 80%) and are centered at the means.

The main results of this study were (1) cows treated with either ASA or MEL tended to have higher type 1/type 2 ratios in the first week after calving compared with PLC cows, (2) regardless of treatment, parous cows had higher type 1/type 2 ratio compared with nulliparous cows, and (3) there was a weak positive correlation between D2Dx scores at 14 ± 3 DIM and average daily milk yield in the first 60 DIM.

During the transition period, dairy cows experience a decrease in immune system functions (Drackley, 1999). The reasons for this immunosuppression during this sensitive time for dairy cows are still unknown. Early studies in mastectomized cows suggest that this impaired immune response may not be solely related to the gestation, calving, or lactation processes, and that instead, authors hypothesized that it could be associated with the metabolic changes that occur during the transition period (Kimura et al., 1999, 2002; Nonnecke et al., 2003). In vitro studies reported that high concentration of NEFA and BHB can negatively affect critical functions of immune cells, such as the oxidative burst in neutrophils and antibody production and secretion in lymphocytes (Lacetera et al., 2004, 2005; Scalia et al., 2006; Ster et al., 2012). Low availability of glucose during this period can also negatively affect the immune system by decreasing proliferation, survival, and function of leukocytes (Ingvartsen and Moyes, 2013; Kvidera et al., 2017). Similarly, low levels of serum calcium can negatively affect the immune system. For instance, Martinez et al. (2014) reported that neutrophils harvested from cows with hypocalcemia have a reduced ability to phagocytize pathogens.

During this period, cows also experience systemic inflammation as a result of trauma related to the parturition process and pathogen contamination in different organs such as the uterus, mammary gland, and intestine (Kuhla, 2020; Mezzetti et al., 2020). This inflammatory process is highly correlated with the physiological challenges mentioned above, and they can exacerbate this response (LeBlanc, 2010; Pascottini et al., 2020), making the immunological response inefficient and wasteful.

Treatment with anti-inflammatory drugs, such as NSAIDs, has been proposed as a strategy to modulate inflammation during the transition period. For instance, Barragan et al. (2020b) reported that multiparous cows treated with ASA after calving had lower concentration of haptoglobin, a pre-acute inflammatory protein used as the gold standard inflammatory biomarker for cattle. Similarly, Jimenez et al. (2023) reported that cows treated with a single dose of ASA 14 before expected calving date had a lower concentration of haptoglobin compared with cows treated with a placebo. This modulation of inflammation may have positive effects on immune system functions. For instance, Pascottini et al. (2020) treated cows with meloxicam, another common NSAID studied for postpartum anti-inflammatory management in dairy cattle, for 4 d from 10 to 13 d after calving and found a tendency for a higher percentage of polymorphonuclear neutrophils, collected at 21 DIM, that were able to engulf beads. Furthermore, the authors reported that the proteolytic degradation of neutrophils after endocytosis was 27% greater in treated cows compared with control cows (Pascottini et al., 2020). In addition, it has been reported that cow treated with NSAIDs after calving had lower incidence of infectious disease, which could be indirectly linked to a better immune response in treated animals. Barragan et al. (2021) found that cows treated with ASA during the first 2 d after calving had lower incidence of clinical metritis and clinical endometritis compared with untreated animals.

A better immunity status is commonly found in older animals as they have been exposed to a wider variety of pathogens, and disease prevention farm management practices such as vaccination, compared with younger animals. A study that assessed immune cell populations in peripheral blood and colostrum of healthy cows in the first days after calving reported that third+ lactation cows had lower concentration of lymphocytes in the peripheral blood compared with primiparous cows, but the concentration of this cells in the mammary gland was higher (Ohtsuka et al., 2010), which could reflect a better immunity at the organ level. In a study that assessed data from 28,230 Holstein cows, authors reported that primiparous cows had higher incidence of some common infectious diseases such as clinical metritis and endometritis, whereas multiparous cows had higher incidence of other infectious diseases such as mastitis and pneumonia (Lean et al., 2023). The negative correlation between SCC and milk yield has been widely reported (Jones et al., 1984; Cinar et al., 2015). The transition period is one of the most susceptible times for cows to develop intramammary infections, characterized by high SCC, due to the risk of milk leakage, and udder pathogen contamination, associated with the dry-off process and the immune depression experienced at this time (Tančin et al., 2018). Therefore, a stronger immune system around parturition could be associated with lower SCC in milk and higher milk yields. Unfortunately, SCC was not collected in this study, but it could be a possible explanation for the observed results. An exacerbated and inefficient inflammatory response during the transition period could also affect milk yield. Kerwin et al. (2022) reported that cows that had higher concentrations of haptoglobin (i.e., ≥0.45 g/L) during the postpartum period produced 492 kg less ME305 compared with cows with lower haptoglobin concentration.

These results suggest that prepartum anti-inflammatory therapies might be associated with a higher shift toward type 1 immunity shortly after calving. Interestingly, higher type 1/type 2 ratios may be associated with higher daily milk yields in the first 60 DIM. Larger studies are needed to identify associations between the D2Dx immunity test and cow health and performance, as well as to assess the applicability of this type of tests in a conventional farm setting.
